# Fabrication of laser printed microfluidic paper-based analytical devices (LP-µPADs) for point-of-care applications

**DOI:** 10.1038/s41598-019-44455-1

**Published:** 2019-05-27

**Authors:** Rajesh Ghosh, Saranya Gopalakrishnan, Rangasamy Savitha, Thiruvengadam Renganathan, Subramanium Pushpavanam

**Affiliations:** Department of Chemical Engineering, Indian Institute of Technology Madras, Chennai, 600036 India

**Keywords:** Lab-on-a-chip, Biosurfaces, Sensors, Chemical engineering, Biomedical engineering

## Abstract

Microfluidic paper-based analytical devices (µPADs) have provided a breakthrough in portable and low-cost point-of-care diagnostics. Despite their significant scope, the complexity of fabrication and reliance on expensive and sophisticated tools, have limited their outreach and possibility of commercialization. Herein, we report for the first time, a facile method to fabricate µPADs using a commonly available laser printer which drastically reduces the cost and complexity of fabrication. Toner ink is used to pattern the µPADs by printing, without modifying any factory configuration of the laser printer. Hydrophobic barriers are created by heating the patterned paper which melts the toner ink, facilitating its wicking into the cross-section of the substrate. Further, we demonstrate the utilization of the fabricated device by performing two assays. The proposed technique provides a versatile platform for rapid prototyping of µPADs with significant prospect in both developed and resource constrained region.

## Introduction

The potential to transform paper into smart microfluidic chips has given way to a number of fluidic applications, involving clinical diagnosis^1–4^, cell biology^5,6^, drug screening^7,8^ environmental monitoring^9–11^, and food safety analysis^12–15^. Over the past decade, the miniaturization of several laboratory analytical functions into small and flexible paper-based chips integrated with immediate response based analysis^16^ have enabled new possibilities in the field of portable and low-cost point-of-care diagnostics^17,18^. However, one key aspect that has prevented the successful commercialization of microfluidic paper-based analytical devices (µPADs) is the utilization of sophisticated and uncommon tools that require substantial technological intervention and human expertise for their manufacture^19,20^. This has caused the field to lag in terms of ease of scale-up and commercialization, even after completing more than a decade since the inception of the first µPAD conceptualized for portable diagnostics by Whitesides and co-workers^21^.

Presently, µPADs are fabricated using several techniques, such as, photolithography^21–23^, wax printing^24,25^, ink jet etching^26,27^ and printing^28^, screen printing^29^, plasma treatment^30^, flexographic printing^31^, CVD^32^, laser treatment^33^, hand held or automated tools^34–38^ etc. While some of these methods like photolithography, plasma treatment and CVD provide good precision and reproducibility^39^, they are expensive and involve complex procedures, making them difficult to be carried out in unfurnished laboratory conditions^20^. On the other hand, low-cost printing techniques such as wax printing and ink-jet printing show good promise in terms of rapid prototyping, but wax printers are not commonly available and ink-jet printers require substantial modification for configuring them to fabricate µPADs^19^. Moreover, patterning of µPADs, using materials such as wax, pose several challenges of flow control of the hydrophobic barrier into the porous structure of the paper^19,40^, causing uncertainty and inhomogeneity. All these challenges necessitate the need for a simpler method to fabricate µPADs.

Herein, we report for the first time, a simple, novel and facile method to pattern microfluidic paper-based analytical devices using a laser printer (LP-µPAD). We employ the inherent precision of a xerography based commercial laser printer to fabricate high-resolution microfluidic devices on paper. The direct printing process renders the automated deposition of hydrophobic toner ink, eliminating human intervention and non-uniformity in fabrication. The approach endorses rapid prototyping that drastically reduces the cost and complexity of fabrication. Additionally, it promotes the WHO recommended ASSURED criteria for point-of-care devices by being affordable, user-friendly and rapid. The facile technique proposed can be used to fabricate LP-µPADs at any point-of-need locations, due to the ubiquitous nature of the tools employed. Further, we show the utilization of the fabricated devices by performing a lateral flow and micro-well spot assay for the quantitative detection of nitrite and *Escherichia coli* respectively. The versatility and simplicity of the fabrication technique described will lead to its wide spread adoption in both developed and resource-constrained settings.

## Results and Discussion

### Patterning of LP-μPADs

**Figure 1:**
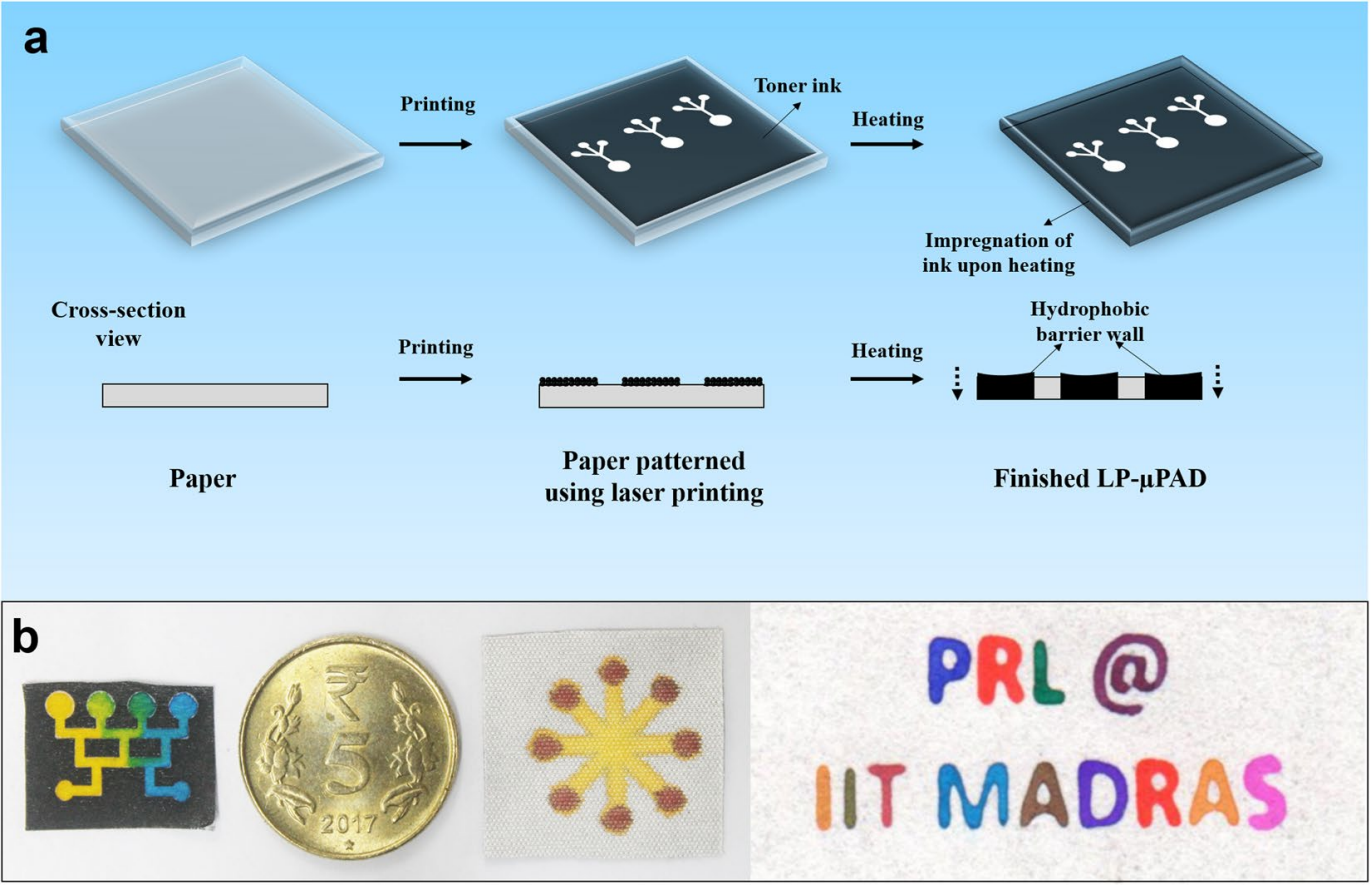
Fabrication of LP-µPAD. (**a**) Schematic representation of the fabrication of LP-µPADs involving just two simple steps: printing using a ubiquitous commercial laser printer, followed by heating of the printed substrate for vertical impregnation of the hydrophobic toner ink from the surface into the entire cross-section of the paper. The impregnation of the toner ink forms permanent hydrophobic barriers that confines the fluid within the demarcated hydrophilic zones. (**b**) Different patterns fabricated to highlight the versatility of the proposed method. Yellow and blue dye mixed in a concentration gradient generator fabricated on tissue paper (see Supplementary Movie 1), a five rupee Indian coin illustrating the size of the devices fabricated and a multiplexed flower patterned LP-µPAD fabricated on polyester-cellulose task wipes, demarcated with multilayer dye. The letters “PRL @ IIT MADRAS” patterned on Whatman No.1 filter paper.

We used the solid toner ink from a laser printer to construct the hydrophobic barriers in the LP-µPADs. The devices were fabricated by two simple steps as shown in Fig. 1a – printing the desired pattern using a laser printer and heating the printed paper to melt the toner ink into the capillary network of the substrate. A pictorial representation of the process is shown in Supplementary Fig. 1. The original toner ink which forms the basis of the hydrophobic barrier, contains a number of components such as styrene acrylate resin (<55%), ferrite (<45%) and wax (<10%))^41^. Upon heating, the composite toner ink binds to the porous capillaries of the cellulose based paper forming stable and permanent hydrophobic barriers in the LP-µPADs. Figure 1b shows different patterns constructed on cellulose based substrates such as tissue paper, task wipes and Whatman No. 1 filter paper, using the present method. Mixing of two different dyes in a concentration gradient generator is shown in Supplementary Movie 1. The ability to construct these well-defined hydrophobic barriers in various substrates, highlights the flexibility of the proposed technique.

In laser printing, the ink is deposited by means of an electrostatic charge coupling between the photoreceptor drum, toner micro-particles and the printing media. This default laser precision technology embedded in the commercial laser printer was utilized for the deposition of the hydrophobic ink, rendering the fabrication of microfluidic devices having high resolution. Moreover, the laser printer employed had a resolution of 600 dots per inch (dpi) which distributed the toner ink uniformly ensuring homogeneity and repeatability of the fabricated device. Inside the laser printer, the ink deposited paper passes through a fusion unit that partially melts the toner particles binding them to the surface. However, this short heat treatment is not sufficient for the complete impregnation of the hydrophobic ink across the thickness of the substrate. Further heating is required to melt the toner ink facilitating its wicking into the fibres of the cellulose based paper.

**Figure 2:**
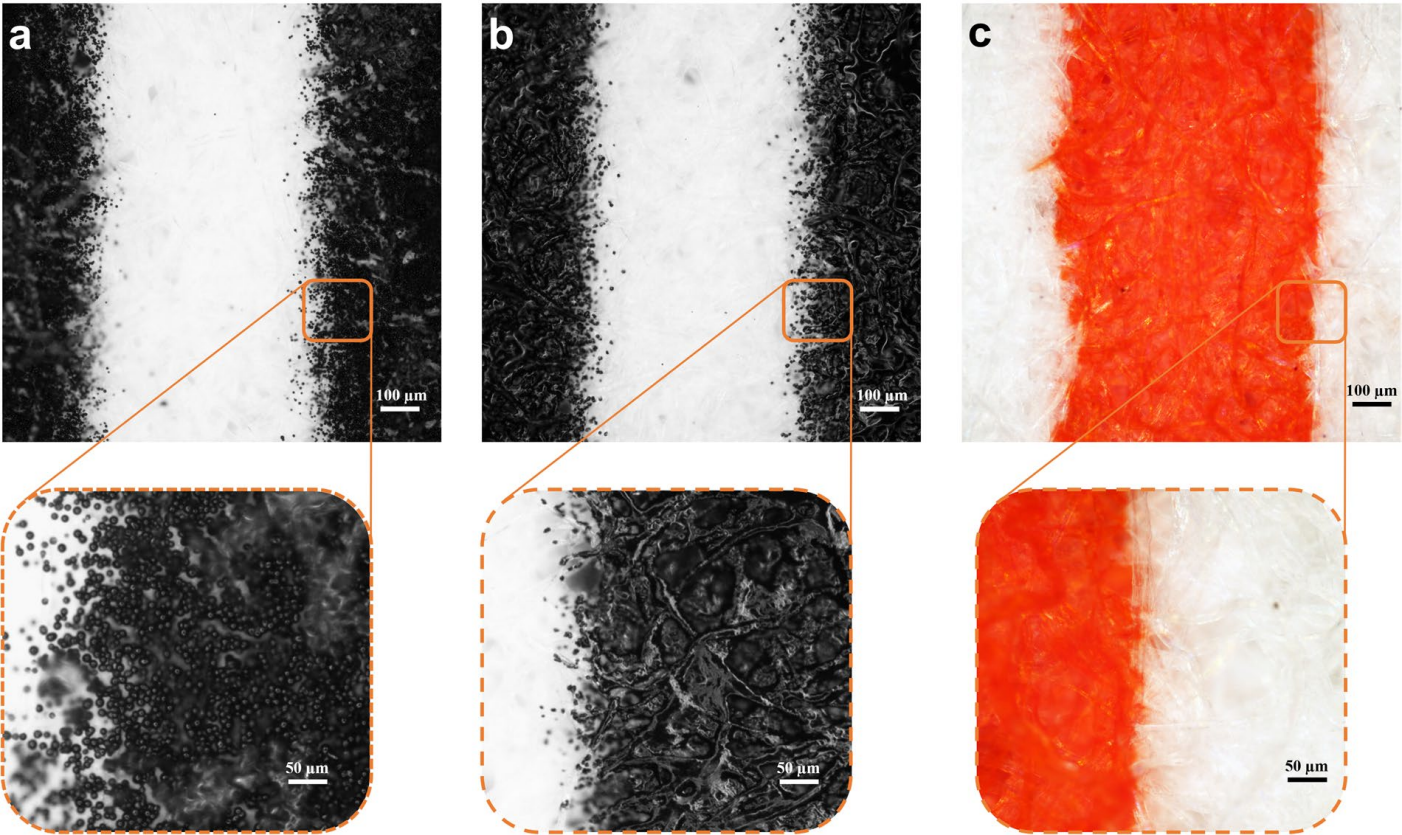
Brightfield microscopic observation of a 500 µm channel surface printed on Whatman No.1 filter paper. The boundary region of the channel was magnified to illustrate the detailed features. (**a**) The surface of the laser printed channel before heating, showing the presence of spherical toner particles. (**b**) Upon heating at 165 °C for 15 minutes, the toner particles melt and impregnate the cellulosic micro-fibres forming a continuous hydrophobic barrier. (**c**) The back side of the channel with the hydrophilic and hydrophobic region distinguished using a red dye.

Figure 2 shows the surface of the printed filter paper as observed under a microscope before and after heat treatment. The printed substrate without heating revealed spherical toner particles deposited on the surface (Fig. 2a). On heating, the particles melted and penetrated the cellulosic micro-fibres of the filter paper across its thickness (Fig. 2b). The black pigment from the toner remain on the top surface, whereas the transparent polymeric resin melts and wicks into the cross-section of the paper. Figure 2c shows the back side of the fabricated channel where the demarcation between hydrophobic and hydrophilic region is shown by wetting using a red dye. This confirms impregnation of the hydrophobic barrier through the entire cross-section of the filter paper. The present method of printing solid toner particles eliminates the difficulties faced using wax as the hydrophobic barrier. The major constraint of using wax based patterning was the difficulty in achieving flow control of the molten wax into the porous capillaries of the paper^40^. On the other hand, toner which is primarily composed of a styrene acrylate based resin, softens at a temperature range of 100–150 °C^42^. This is evident from the dynamic scanning calorimetric data shown in Supplementary Fig. 2. Thus, controlled heating of the toner coated paper at an elevated temperature of 165 °C, above the softening point of the polymer, transforms it to a viscous fluid that wicks gradually into the paper. Further, we observed that when the printed papers were heated at 100 °C for more than an hour, no hydrophobic barrier was formed. This confirmed: a) the wax component (melting point: 60–70 °C) of the toner did not contribute to the hydrophobicity of the µPADs, b) 100 °C was not sufficient to melt the polymeric resin and make it wick along the capillaries of the paper. Another advantage of using toner ink to form hydrophobic boundaries is that, the fabricated devices possessed high thermal stability. This is due to the high softening point of the polymer which is above 100 °C. We investigated the barrier stability by incubating the LP-µPADs at 100 °C for 60 minutes. No significant change in the wetting behaviour of the hydrophilic region was observed which confirmed that the stability of the barrier was retained even after exposure to high temperature. The wicking of the hydrophobic polymer to form permanent barriers could be controlled by varying the heating time. The patterned devices were heated for 5–120 minutes and characterized to determine the optimum conditions.

### Hydrophobicity

To ensure good liquid confining properties in the fabricated LP-µPADs, it was necessary to construct a barrier that exhibited strong hydrophobic characteristics. The degree of hydrophobicity was evaluated by measuring the contact angle of a sessile water droplet on the hydrophobic region of the fabricated device. The barrier constructed showed a high hydrophobicity with an average contact angle of 131.2 ± 2° for different heating periods. The maximum relative standard deviation (RSD) in the measurements was 1.45% (n = 5) (Supplementary Fig. 3). Past investigations using different fabrication methodologies and barrier materials, reported contact angle ranging from 110~128°^32,37,43,44^. This showed significant promise towards the use of composite toner ink for patterning hydrophobic barriers in µPADs comparable to other conventional materials like SU-8 resin^45^, wax^46^ and PDMS^36^.

### Surface topography

**Figure 3:**
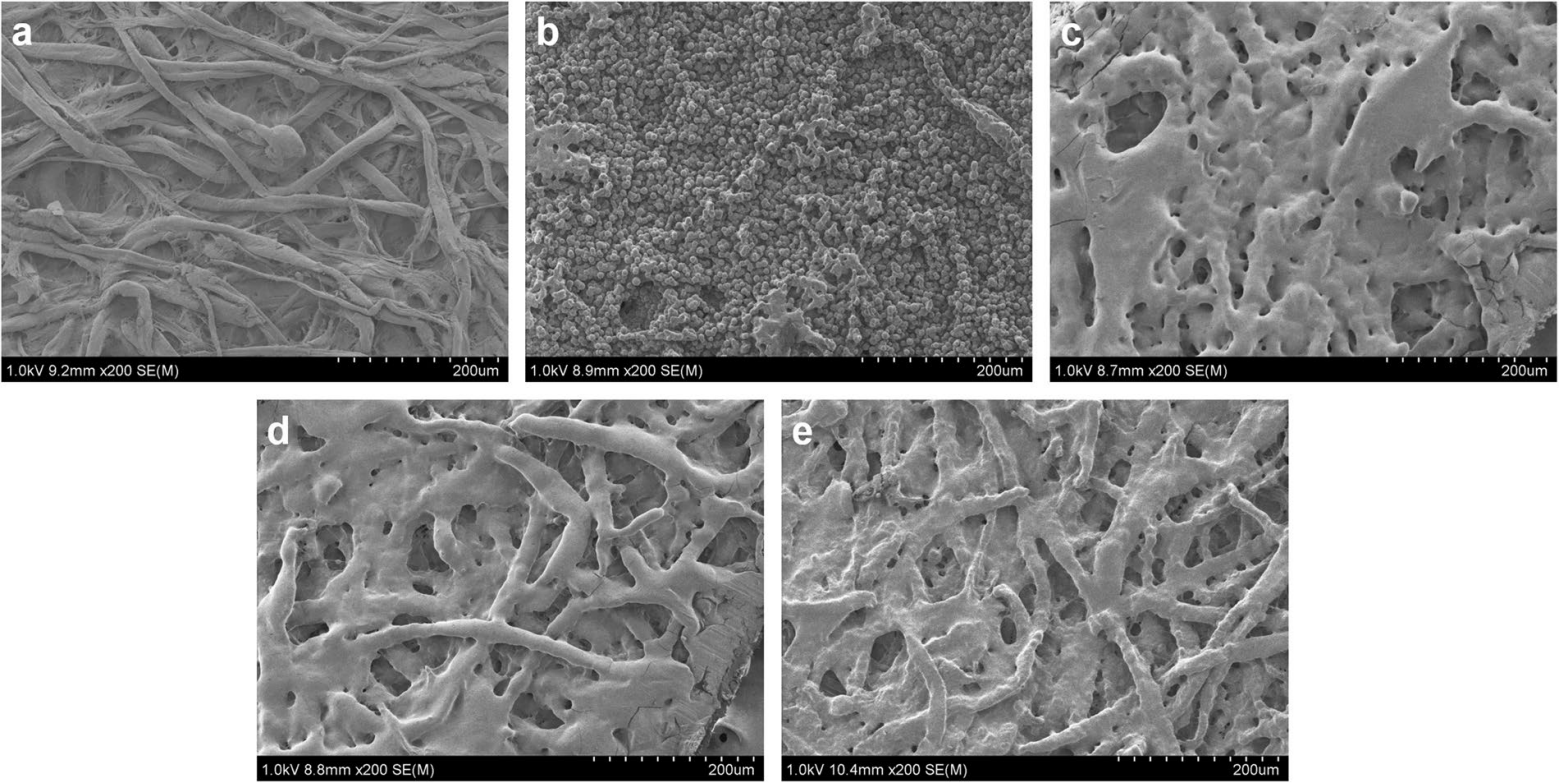
SEM micrograph depicting the effect of heating time on the wicking action of the toner. (**a**) Surface of the filter paper before printing. (**b**) Printed filter paper, showing deposition of spherical toner particles. (**c**) Printed filter paper heated for 5 minutes showing large chunks of molten toner coated on the surface. (**d**) Printed paper heated for 15 minutes showing a decrease in the amount of toner deposited on the surface. This is due to wicking action of the viscous polymer across the thickness of the filter paper to the other end. (**e**) Printed filter paper heated for 60 minutes showing thin coating of toner on the cellulosic fibres. The decrease in the toner on the surface is due to the continuous wicking action across the capillary network of the paper.

Figure 3 shows the surface topography of the Whatman No.1 filter paper examined using a scanning electron microscope (SEM) before printing, after printing and for different heating periods of the printed paper. Figure 3a depicts the surface of the filter paper. Upon printing, the spherical toner ink particles (2–10 µm) are deposited on the top of the filter paper as shown in Fig. 3b. The change in the surface morphology of the toner coated paper, with increase in the heating duration was observed in Fig. 3(c–e). The amount of toner coated on the surface of the substrate reduces with increase in heating time, exposing the cellulosic fibres. This confirms the wicking action of the molten polymer into the cross-section of the filter paper.

### Liquid confinement

**Figure 4:**
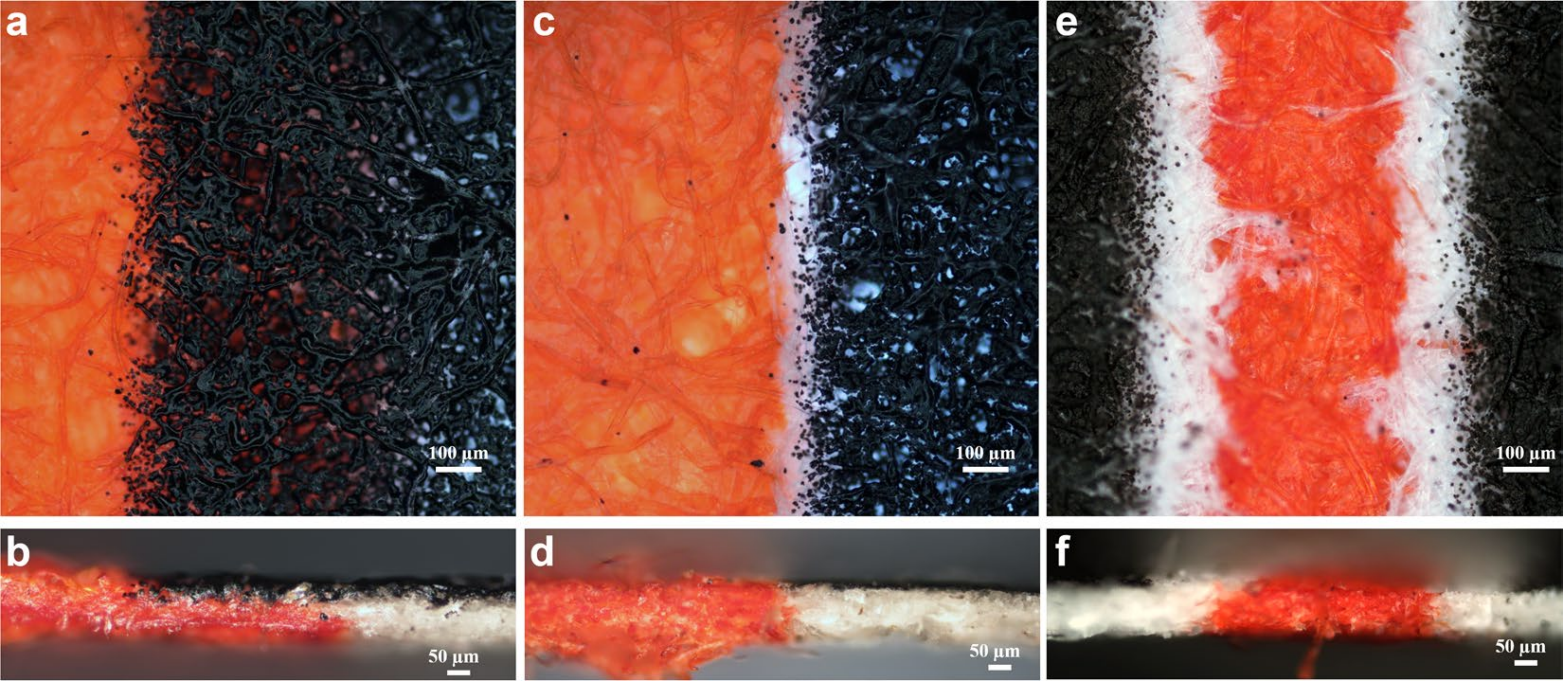
Effect of heating time on the performance of a 700 µm channel for fluid confinement. (**a**) Front view of the insufficiently heated channel for less than 5 minutes, the dye solution readily leaked into the hydrophobic zone (n = 10). (**b**) Cross-sectional view of the leaked channel. (**c**) Channel heated for an optimum duration of 15 minutes, the dye is successfully confined within the hydrophilic zone (n = 15). (**d**) Cross-sectional view of the successfully confined channel. (**e**) Front view of an overheated channel, the viscous hydrophobic polymer wicked into the hydrophilic zone, reducing the hydrophilic area (n = 10). (**f**) Cross-sectional view of the partially blocked channel.

Next, the role of heating duration on the ability of the device for fluid confinement was studied. A drop of the red food colorant was introduced in the channel and the spread of the colour was observed under the microscope. Figure 4 shows the section of a 700 µm channel revealing the spread of the dye, in the patterned paper, heated for different durations. Figure 4a,b corresponds to the front and cross-sectional view of the channel heated for 5 minutes. It can be observed that the dye leaks from the hydrophilic region into the hydrophobic barrier section due to incomplete penetration of the toner across the cross-section of the substrate (n = 10). Figure 4c,d shows channel heated at 165 °C for 15 minutes, showing good liquid confinement within the hydrophilic region (n = 15). Thus, a heating duration of 15 minutes was found optimal for both leak-proof and sharp boundaries between the hydrophobic and hydrophilic regions. When the patterned paper was heated for longer durations, the hydrophobic polymer wicked into the hydrophilic region reducing the flow area. Figure 4e,f shows the case of an overheated device, where the width of the channel was reduced from 700 µm to 400 ± 50 µm due to lateral flow of the polymeric ink into the hydrophilic zone (n = 10).

### Channel resolution and minimum barrier width

**Figure 5:**
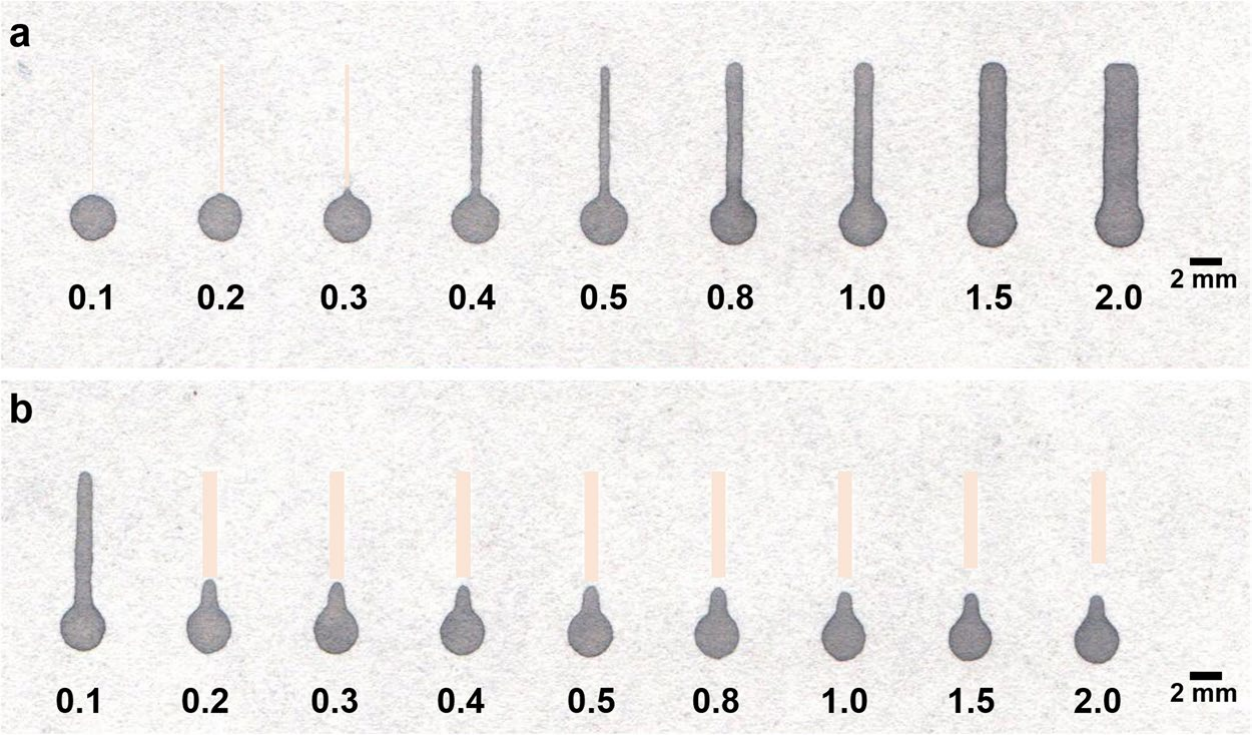
Resolution of the current method for fabricating LP-µPADs as observed on the backside of the patterned paper. (**a**) Hydrophilic channel of varying widths (0.1–2 mm) along with a 3 mm circular reservoir fabricated by heating at 165 °C for 15 minutes to show the resolution of the laser printing method (hydrophilic channel zones are colour modified for visual demarcation). A minimum hydrophilic channel of 415 ± 35 µm was obtained (n = 11; %RSD of 8.34). (**b**) Barriers having varying widths fabricated across a 1 mm hydrophilic channels, by heating at 165 °C for 15 minutes to determine the minimum barrier width required to arrest flow of liquid. A minimum printed line of 200 µm was necessary to restrict flow (n = 10 and success rate of 60%).

To determine the resolution of the current method, channels of different widths (0.1–2 mm) were tested by conducting flow studies (n = 11) using dye solution, for different heating durations. For low heating time, the dye leaked through the channels (Supplementary Fig. 4a), while for higher heating time, the flow of dye was obstructed by the lateral flow of the hydrophobic polymer into the hydrophilic area, affecting the resolution of the channel (Supplementary Fig. 4c,d). A heating time of 10 minutes (Supplementary Fig. 4b) was sufficient for the complete penetration of the hydrophobic barrier to produce leak-proof channels. But the interface between the hydrophobic and hydrophilic regions was not sharp. A heating time of 15 minutes was found to be optimum for which, a minimum hydrophilic channel width of 415 ± 35 µm could be obtained with RSD of 8.34% (Fig. 5a). Further, we noted that the width of the printed channels was more than the preset value by ~50 µm. This is attributed to the uneven distribution of toner at the edges of the printed region arising from the low resolution of the 600-dpi printer used in this work. However, this was overcome by heating the printed channels, which controlled the lateral wicking of the hydrophobic polymer.

To determine the minimum barrier width required to restrict the liquid flow, barriers of different widths were fabricated across a 1 mm hydrophilic channel using the optimum heating conditions. A 3 mm circular inlet was provided at one end of each channel and the dye solution was introduced. A successful hydrophobic barrier was defined as one which could prevent the incoming flow of dye from the channel reservoirs, into the remaining section of the channel. Results show that a minimum printed barrier of 200 µm was necessary to impede the flow of the dye (n = 10) (Fig. 5b). However, barrier widths of 200 µm and 300 µm were successful in only 60% and 70% of the experiments respectively. For barrier widths of 400 µm and above, the success rate of the devices in confining fluid flow to the remainder of the hydrophilic portion of the channel was 100%. A detailed comparison of the current fabrication method with past techniques is presented in Supplementary Table 1.

Hydrophilic channels having width less than 400 µm could not be fabricated due to limitations of the aspect ratio of the filter paper substrate. However, the resolution of the channels obtained could be improved by choosing a thinner substrate, but it was not considered as an objective of this work. One important aspect that should be taken into consideration while fabricating very narrow channels (≤1 mm) by using hydrophobic barriers is the reduction of fluid flow velocity with decrease in channel width^47–49^. Use of very narrow channels increases the travelling time of fluid due to inverse surface tension effects^48^, thereby increasing the response time.

### Stability against surfactants and solvents

**Figure 6:**
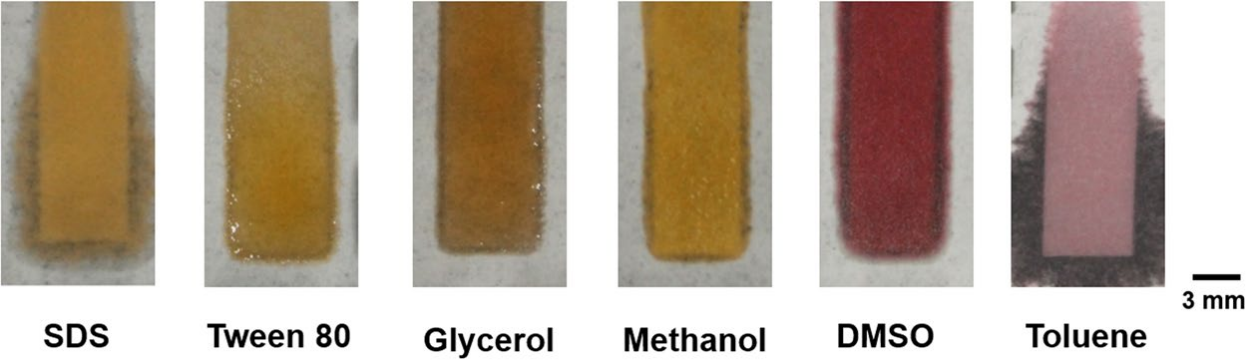
Flow of different surfactant and solvents across 5 mm width and 20 mm length channel showing the stability of LP-µPADs against different solvents.

Figure 6 shows the stability of the toner-based barriers when exposed to various solvents and surfactant solutions. The barriers were susceptible to toluene and sodium dodecyl sulfate (SDS) resulting in complete leakage outside the hydrophilic channels. While toluene leaks from the channel almost immediately upon addition, the leakage of SDS occurred over time (10 minutes). It was interesting to note that the barriers were resistant to Tween 80, glycerol, methanol and dimethyl sulfoxide (DMSO). Thus, the LP-µPADs could successfully resist the attack of solvents and surfactants like glycerol, methanol, DMSO and Tween 80 showcasing the robust compatibility of the devices with a variety of solvents.

### Assay demonstration

To show the utilization of the proposed fabrication method, LP-µPADs were demonstrated by performing a nitrite assay and an enzymatic detection of *E*. *coli*. species. Nitrite, a well-known metabolite and a marker for urinary tract infection^50,51^ and *E*. *coli*, a common food borne pathogen^52^ and a causative agent of urinary tract infection, were chosen as the analytes. The ability to perform these routine tests using the fabricated LP-µPADs increases their applicability of these devices for day-to-day use.

### Nitrite assay

**Figure 7:**
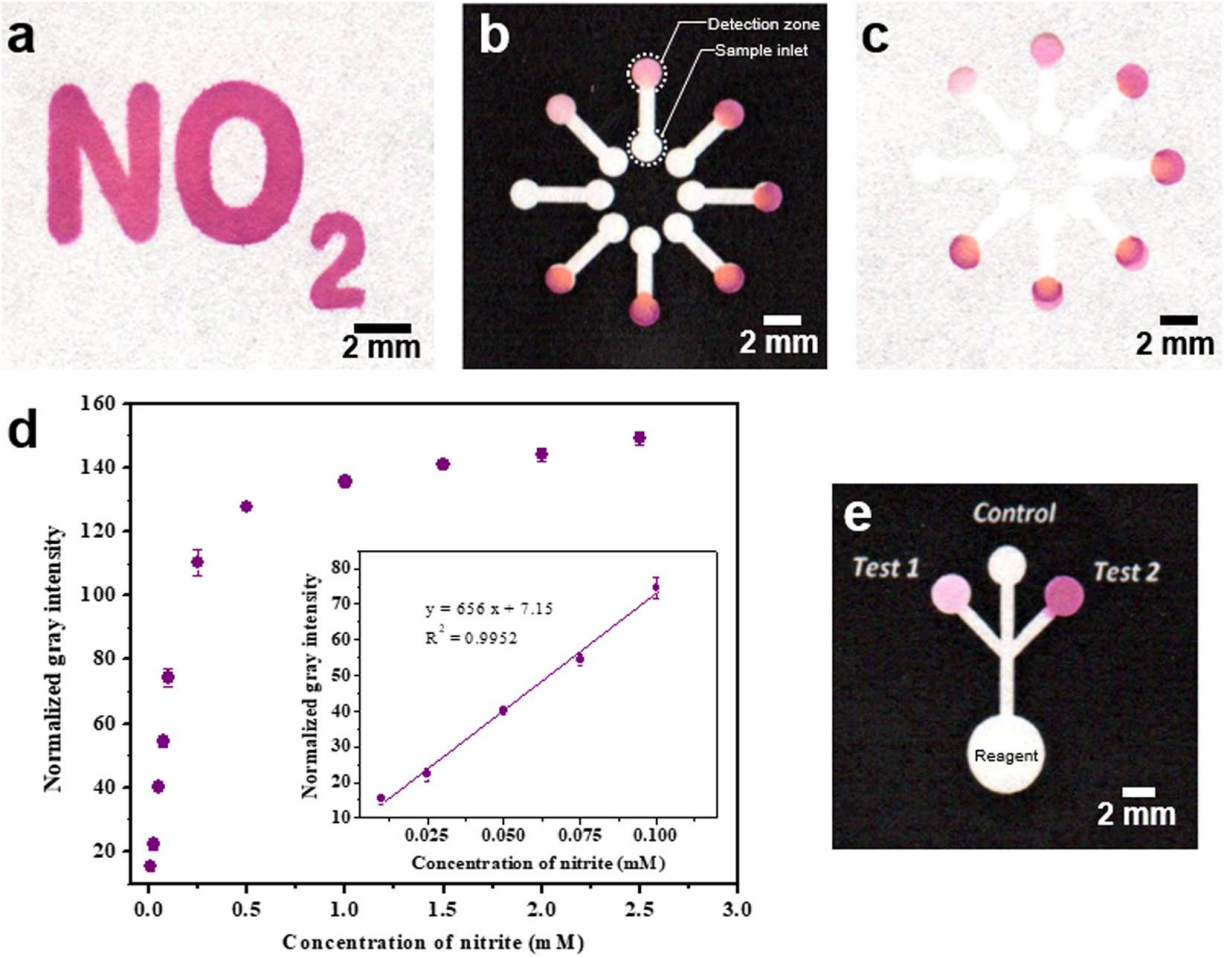
Nitrite assay. (**a**) A ‘NO_2_’ patterned LP-μPAD that changes colour upon sensing nitrite. (**b**) Front side of a flower type lateral flow multiplexed LP-μPAD with 8 detection zones and sample entry points; the samples were added to the reservoirs located at the interior of the design and allowed to flow to the detection zones in the periphery. (**c**) Back side of the flower type lateral flow LP-μPAD. (**d**) Variation of the mean normalized gray scale intensity measured on the back side of the LP-µPAD using ImageJ with the concentration of nitrite, and a linear calibration chart obtained in the range of 0.01–0.1 mM of nitrite (n = 3). (**e**) A LP-μPAD fabricated for on-site sampling of nitrite in artificial urine.

Figure 7a shows a ‘NO_2_’ symbol patterned LP-µPAD for the detection of nitrite. The Griess reagent prepared in 80% methanol was added to the pattern and allowed to flow. The hydrophobic barrier was stable against the organic solvent, confining it within the hydrophilic detection zone. The nitrite containing sample was loaded onto the pattern and a pink colour developed, confirming the presence of nitrite. Figure 7b shows a flower shaped lateral flow LP-µPAD used for generating the calibration plot with known standard solutions. Each segregated petal corresponds to one concentration of the standard solution. The variation in colour for different concentration of the standard solution is clearly observed from the front as well as back side of the device as shown in Fig. 7b,c. The colorimetric signals were measured in terms of red, green and blue intensity, from the back side of the LP-µPAD, using ImageJ software^53^. The colour intensity was notably higher in the backside of the device. Figure 7d shows the variation of mean normalized gray intensity with the concentration of nitrite. A linear calibration chart was obtained in the range from 0.01 to 0.1 mM of nitrite (n = 3). A limit of detection (LOD) of 7 µM was obtained (95% confidence interval, 6.2 to 7.6 µM). Multiple nitrite samples of different concentrations could be measured using a single microfluidic paper chip without cross-contamination. This can also be extended to measuring multiple analytes in each branch of the flower type LP-µPAD design. Further, the detection zone could be minimized without loss in resolution which increased the chemical loading per unit surface area of the device, enhancing the colorimetric signal obtained. The LP-µPADs fabricated were also used to quantitatively determine the concentration of nitrite in spiked artificial urine samples. Figure 7e shows the LP-µPAD used for the detection of nitrite in artificial urine samples of spiked concentrations of 0.01 mM (Test 1) and 1 mM (Test 2). The device was activated by adding the reagent in the central reservoir, and the individual samples were added in each micro-spot for analysis. The device showed good sensitivity for the detection of nitrite even at trace concentrations in the artificial urine samples.

### Live bacterial cell detection

**Figure 8:**
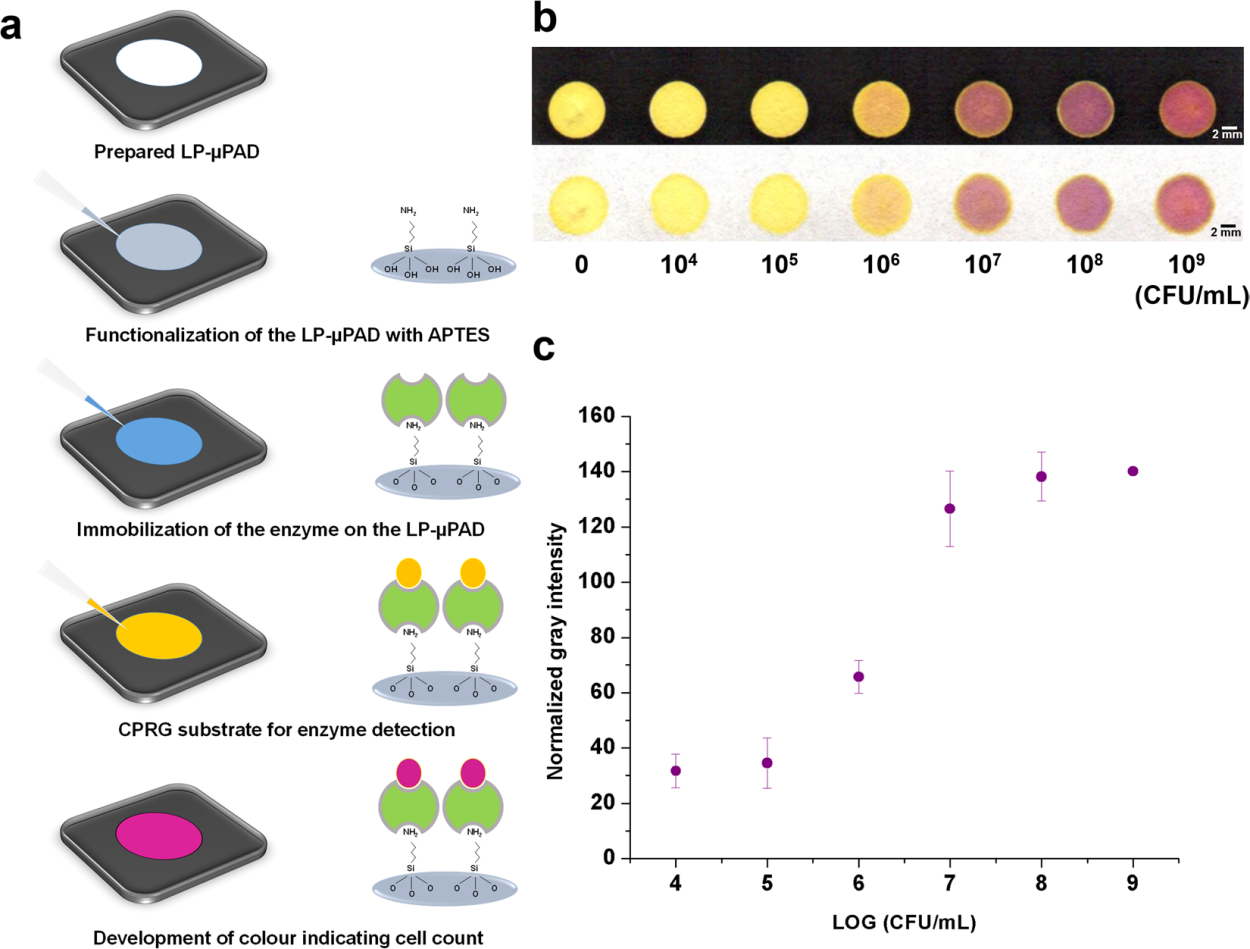
Micro-well spot assay for the detection of live bacterial cell. (**a**) Protocol for detection of live bacterial cell of test species, *E*. *coli*. (**b**) Front and back side of the micro-well LP-µPAD showing a clear change in colour with the increase in the bacterial cell count. (**c**) Variation of mean normalized gray intensity with colony forming unit (CFU)/mL of *E*. *coli* (n = 3).

The cell count of *E*. *coli*., a gram-negative bacterium was determined by the procedure shown in Fig. 8a. The LP-µPADs were functionalized with APTES to enhance the immobilization of the β-galactosidase enzyme from the lysed *E*. *coli*., on the entire surface of the micro-well spot. The APTES underwent hydrolysis in the aqueous environment to form silane groups binding covalently with the hydroxyl functional groups of the cellulose based paper^54^. The amine groups on the APTES functionalized cellulose substrate conjugate with the COOH group of the enzyme immobilizing them on the paper disks. Chlorophenyl red β-galactopyranoside (CPRG) was added to the enzyme coated surface which resulted in colour change from yellow to red-violet, indicating the presence of *E*. *coli*. Using the protocol, LP-µPADs provide a rapid platform for screening live bacterial samples by reducing the diagnosis time compared to standard plating protocols (from ~18–48 hours to ~4–5 hours)^52^. The three-dimensional matrix and high surface area of the device reduced the reaction time producing a fast colorimetric response. Additionally, only 2.2 µL of the CPRG substrate was sufficient for a single assay. Figure 8b shows the gradient in colour intensity obtained for different population of the cells both in the front and back of the LP-µPAD. Figure 8c shows the normalized gray intensity for the different cell counts of the species (n = 3). The lowest concentration of *E*. *Coli*. that could be measured using the LP-µPAD was 10^4^ CFU/mL. A noteworthy advantage of the current device is the reduction in diagnosis time which was due to the pre-concentration of the intracellular enzyme immobilized on the micro-well LP-µPADs. Further, the choice of utilizing a lactose broth for enrichment of the cells^55^ and its pre-concentration resulted in faster screening of samples. Based on this demonstration, the LP-µPADs can also be modified to detect other pathogenic organisms such as *Salmonella typhimurium* and *Listeria monocytogenes*^52^, with faster screening of samples.

## Conclusions

We present a simple, affordable and universally accessible platform for rapid fabrication of LP-µPADs which find application in a number of scientific disciplines, especially in the fabrication of low-cost analytical tools. The key advantage is that any laser printer which uses a hydrophobic polymer as one of the major constituent in the toner can be readily used for the preparation of the LP-µPAD, without modifying any factory settings. The micro-fabrication procedure can be used to create any pattern or fluidic architecture and can be completed from concept to final LP-µPADs within minutes. This was demonstrated by fabricating a wide variety of patterns on different cellulose based porous substrates such as Whatman No.1 filter paper, polyester-cellulose task wipes and tissue paper. The present method circumvents the requirement of any clean-room facilities, fabrication masks, expensive or harsh chemicals. Moreover, the fabricated devices are highly reproducible, thermally stable and compatible with organic solvents such as methanol and DMSO.

In conclusion, we show a simple, inexpensive, easily scalable rapid prototyping technique for the fabrication of LP-µPADs. Our work shows a promising direction towards the large-scale manufacture of these low-cost, disposable diagnostic sensors in both developed and underdeveloped region. Overall, the method is a potential step closer to the direct printing of microfluidic paper-based lab-on-a-chip devices analogous to the fabrication of printed microelectronic chips.

## Methods

### Materials and equipment

Whatman No. 1 filter paper 46 × 57 cm (GE Healthcare Life Sciences, UK) was used to fabricate the LP-µPADs. Tissue paper, cellulose-polyester based task wipes and coloured dyes were procured from local vendors. A monochrome laser printer (HP LaserJet Pro P1606dn) was used to print hydrophobic barriers on the substrates with original toner cartridge (CE 278A) having a composition of styrene acrylate resin (<55%), ferrite (<45%) and wax (<10%). Citric acid anhydrous, Sulfanilamide, N-(1-Napthyl) ethylenediamine, dimethyl sulfoxide, Tween 80, sodium dodecyl sulfate and sodium nitrite were obtained from Sigma Aldrich, India. Toluene and methanol were obtained from Merck, India. Sunset yellow dye and nile red were obtained from Spectrochem, India. Chlorophenyl red β-galactopyranoside (CPRG), lyophilized bovine serum albimin, N-(2-Hydroxyethyl) piperazine-N′-2-ethanesulfonic acid (HEPES) and sodium hydroxide was procured from Sigma Aldrich, India and used to prepare substrate for live cell detection. (3-Aminopropyl) triethoxysilane (APTES) obtained from Sigma Aldrich, India was used to functionalize the LP-µPAD to bind the enzyme to the surface. *Escherichia coli* (ATCC 25922 culture) strain was procured from American Type Culture Collection, USA. Peptone from animal tissue, lactose and beef extract were obtained from HiMedia, India and used to prepare the enrichment broth for live organisms.

### Fabrication of LP-µPADs

The schematic of the operational steps for fabricating LP-µPAD is shown in Fig. 1a and Supplementary Fig. 1. The desired pattern was prepared using Microsoft PowerPoint 2016 software and the hydrophobic barrier was printed on Whatman No. 1 filter paper (unless otherwise mentioned) using the laser printer. All the designs were printed with maximum resolution and highest print quality settings. Next, the printed paper was heated uniformly in an oven at 165 °C to impregnate the paper with toner ink. After the impregnation of the hydrophobic barrier into the thickness of the paper by heating, the LP-µPADs were ready to use. Apart from Whatman No.1 filter paper, devices were also fabricated out of tissue paper and task wipe cloth to extend the utilization of the current technique. The Whatman filter papers and cellulose based task wipes were cut into A4 size (21 × 29.7 cm) that could be conveniently supplied to the printer. The tissue paper and task wipes were attached to a standard A4 paper using adhesive tape before feeding to the printer to prevent folding inside the printer.

### Characterization of the LP-µPAD

The paper substrates before printing, after printing and after heating for different periods were subjected to various characterization studies. The surface characteristics were observed using a bright field microscope (Nikon ECLIPSE LV100ND) and scanning electron microscope (HR-SEM, Hitachi S4800). The wetting behaviour was characterized by measuring the contact angle of a 5 µL sessile water droplet placed on the barrier region of the fabricated device. A drop shape analyser was used to measure the contact angle on the hydrophobic region (KRÜS DSA256). The liquid confining ability of the fabricated LP- µPADs were studied by placing a drop of red dye and observing its spread under the microscope. To evaluate the resolution of the current method, channels of different widths (0.1–2 mm) with a 3 mm circular reservoir inlet were fabricated. A drop of dye solution was introduced in the reservoir section and the spread of the colour was observed on the back side of the fabricated LP-µPADs. To evaluate the stability of the toner-based hydrophobic barrier, 15 µL of different surfactant solutions and solvents were added into the channel of width 5 mm and length 20 mm. The test solutions were 1% (w/v) sodium dodecyl sulfate, 1% (w/v) Tween 80, methanol, dimethyl sulfoxide, toluene and glycerol. The concentration of the surfactants used were well above their critical micelle concentration. Sunset yellow dye and nile red were added to the solutions for visualization of wicking action in the channels. The barrier integrity test with different solvents was repeated over five independent trials with consistent observations.

### Nitrite assay

A flower type lateral flow LP-µPAD with multiple detection zone and sample entry points was fabricated by laser printing followed by heating for 15 minutes at 165 °C (Fig. 7b). The Griess colorimetric reaction was used to determine the total nitrite content in aqueous solutions and artificial urine samples. The diazotization of sufanilamide in the presence of nitrite and the subsequent azo coupling reaction with n-(1-napthyl) ethylenediamine gives a pink colour (azo dye), confirming the presence of nitrite^56^. 0.25 µL of the reagent comprising of 50 mM sulfanilamide, 330 mM citric acid and 10 mM n-(1-napthyl) ethylenediamine dissolved in 80% methanol was introduced into each detection zones of the LP-µPAD (Fig. 7b). The device was allowed to air dry for ~2 minutes. Following which, 1.5 µL aliquots of standard solutions (0.01–2.5 mM) were introduced in the respective sample entry zones. A pink colour developed at the corresponding detection zones and its intensity stabilized in 5 minutes. The device was scanned using a desktop scanner and the backside of the entire detection zone was analyzed to obtain the red (R), green (G) and blue (B) intensity using ImageJ software. The normalized gray intensity was calculated using equation 1.

1 $$Normalized\,gray\,intensity=255-(0.2126\,{\rm{R}}+0.7152\,{\rm{G}}+0.0722\,{\rm{B}})$$

The assay was repeated by conducting three independent experiments and the mean normalized gray intensity of the detection zone is reported in Fig. 7d. A calibration chart was obtained between the mean normalized gray intensity and concentration of the analyte. Further, the nitrite content in spiked artificial urine samples were quantitatively determined using a lateral flow LP-µPAD. For this, the reagent was first coated in the entire hydrophilic zone, following which 1 µL of the individual test samples were added in the respective detection zones with blank water as control.

### Live bacterial cell detection

An array of 6 mm circular well type LP-µPADs was fabricated for carrying out the enzymatic assay by laser printing and heating at 165 °C for 15 minutes (Fig. 8b). *Escherichia coli* (ATCC 25922) was used as a test organism to demonstrate the detection of live pathogenic cells in the fabricated LP-µPADs. The strain was sub-cultured in nutrient agar at 37 °C for 24 hours before use. Different concentrations of bacterial cells: 10^4^, 10^5^, 10^6^, 10^7^, 10^8^, 10^9^ CFU/mL were inoculated in an enrichment broth and incubated in an orbital shaker at 37 °C for three hours. Simultaneously, inoculum was plated in nutrient agar medium to estimate the bacterial cell concentration by colony count method. The enrichment broth consisted of lactose (5 g/L), peptone from animal tissue (5 g/L), beef extract (3 g/L). A lactose broth (10 ml) was chosen to stimulate the expression of the β-galactosidase enzyme. The enriched culture was centrifuged at 4 °C and 12.5 relative centrifugal force (RCF) for 10 minutes to further concentrate the cells into 1 ml. The remnant was decanted and the concentrated cells were lysed by sonication at 40% amplitude, 0.5 s pulse rate for 20 seconds to release the intracellular target enzyme into the bulk solution. Chlorophenyl red β-galactopyranoside (CPRG) which is an analogue for β-galactoside was chosen as the chromogenic substrate. The substrate (yellow) produced a red-violet colour in the presence of the enzyme. The well spots on the LP-µPAD were functionalized with 0.85 µL of 5% APTES. The functionalized LP-µPAD was loaded with 14 µL of the concentrated and lysed bacterial culture. The strip was allowed to dry at 37 °C for 30 minutes. Following this, 2.2 µL of 3 mM CPRG stain dissolved in 0.1 mM HEPES buffer at pH 7.5 and complemented with 0.1% bovine serum albumin (BSA) was added to the micro-well spots. A red-violet colour developed almost immediately and the intensity stabilized within 10 minutes, following which the device was scanned and analysed to determine the normalized gray intensity (similar to method described earlier), indicative of the enzyme concentration. The live cell count was directly correlated with the concentration of the enzyme present in the enriched phase.

## Supplementary information

Supplementary Information

Supplementary Movie 1

## Data Availability

The datasets generated during this work will be made available by the corresponding author upon reasonable request.
