# Supplementary material for: Fabrication of laser printed microfluidic paper-based analytical devices (LP-µPADs) for point-of-care applications
Source: Sci Rep. 2019 May 27;9:7896. doi: 10.1038/s41598-019-44455-1 (PMC6536539; doi:10.1038/s41598-019-44455-1)
Supplement: Supplementary file 1 — Supplementary Information [file 41598_2019_44455_MOESM1_ESM.docx]

**Fabrication of laser printed microfluidic paper-based analytical devices (LP-µPADs) for point-of-care applications**

Rajesh Ghosh^1^, Saranya Gopalakrishnan^1^, Rangasamy Savitha^1^, Thiruvengadam Renganathan^1^, Subramanium Pushpavanam^1*^

**^1^**Department of Chemical Engineering, Indian Institute of Technology, Madras, Chennai, 600036, India

*Correspondence and request for materials should be addressed to S.P. (E-mail: spush@iitm.ac.in)

**Supplementary Information**

**Supplementary Figure 1.** Step by step protocol for fabrication of LP-PADs, as described in this work, using commonly available inexpensive tools. **a** Design of the device to be fabricated using any drawing software in a computer. **b** A laser printer is used to print the designed pattern on the paper substrate. **c** The printed paper is heated in an oven at 165 °C that creates vertical hydrophobic walls made of toner ink. **d** LP-µPAD designed is ready for use in just a few minutes.

**Supplementary Figure 2.** Differential scanning calorimetric (DSC) analysis for the toner ink powder. The thermogram reveals an endothermic event at 60-70 °C, indicating the melting of wax. Further, the downward trend in heat flow from 100-150 °C shows the softening of the polymer with increase in temperature. The LP-µPADs were fabricated by baking at 165 °C.

**Supplementary Figure** **3.** Contact angle of a water droplet on the patterned region of LP-µPAD. **a** A sessile water droplet on the surface of a Whatman No.1 filter paper which was hydrophobized by laser printing followed by heating at 165 °C for 15 minutes. **b** Variation of contact angle with respect to heating time (n=5) (relative standard deviation (RSD) ≤ 1.45%).

**Supplementary Figure** **4.** The effect of heating time on the resolution of the fabricated channels at 165 °C. Figure shows back side of the patterned paper with 3 mm circular reservoirs and channels of different width (0.1-2 mm). The dye was added to the circular reservoirs and allowed to flow in the channels (hydrophilic channel zones are colour modified for visual demarcation). **a** For 5 minutes of heating time, the toner ink did not wick completely across the cross-section of the paper, causing the dye to leak into the hydrophobic zone (n=11). **b** For 10 minutes of heating, the spread of the dye was more than the printed width of the channels (n=11). **c** For heating duration of 30 minutes, the flow in 0.8 mm channel is restricted due to lateral wicking of the hydrophobic polymer into the hydrophilic flow area (n=11). **d** For 60 minutes, blockage occurs even in 1 mm channels (n=11).

**Supplementary Table 1.** Comparison of laser printing technique for making µPADs with previous reported fabrication techniques

| **Fabrication techniques** | **Minimum hydrophobic barrier (µm)** | **Minimum hydrophilic channel (µm)** | **Advantages** | **Disadvantages** | **Reference** |
| --- | --- | --- | --- | --- | --- |
| Wax printing | 850±50 | 561±45 | - Simple and rapid method, - Supports mass production | - Low resolution, - Hydrophobic barriers unstable at high temperatures - Requires expensive wax printers which is a rare commodity^13^ - Unstable against organic solvents^14^ | Carrilho *et al.* 2009^15^ |
| Inkjet printing | 302 | 590 | - Mass production - Inexpensive printers - High resolution - Compatible against cell lysing agents - Direct printing of reagents | - Needs modification and improved inkjet printers - Requires multiple printing steps | Li *et al.* 2010^16^, Wang *et al.* 2014^14^ |
| Photolithography | 186±13 | 248±14 | - High resolution | - Requires expensive and toxic consumables - Complex and multiple step procedure - Requires masks for each pattern | Martinez *et al.* 2008^17^ |
| Laser treatment | 62±1 | 62±1 | - High resolution | - Expensive - Not suitable for scale-up | Chitnis *et al.* 2011^18^ |
| Ink-jet etching | - | - | - Low-cost - Direct printing of reagents | - Needs modification and improved inkjet printers - Requires multiple printing steps | Abe *et al.* 2010^19^ |
| Screen printing | 1300±104 | 650±71 | - Uses easily available materials - Simple steps | - Customized masks required - Low resolution | Dungchai *et al.* 2011^20^ |
| Laser Printing | 415±35 | 200 | - Low-cost easily available printer - High resolution - Simple and rapid method - Supports mass production - Hydrophobic barriers stable at high temperatures - Stable against methanol, DMSO, glycerol and Tween 80 | - Unstable against toluene and SDS | *Current work* |
